# Vitamin D, chronic pain, and depression: linear and non-linear Mendelian randomization analyses

**DOI:** 10.1038/s41398-024-02997-7

**Published:** 2024-07-04

**Authors:** Emily Bassett, Eva Gjekmarkaj, Amy M. Mason, Sizheng Steven Zhao, Stephen Burgess

**Affiliations:** 1 MRC Biostatistics Unit, University of Cambridge, Cambridge, CB2 0SR UK; 2 Department of Public Health and Primary Care, University of Cambridge, Strangeways Research Laboratory, Cambridge, CB1 8RN UK; 3 British Heart Foundation Cardiovascular Epidemiology Unit, Department of Public Health and Primary Care, University of Cambridge, Cambridge, CB2 0BD UK; 4 Victor Phillip Dahdaleh Heart and Lung Research Institute, University of Cambridge, Cambridge, CB2 0QQ UK; 5 Centre for Musculoskeletal Research, Division of Musculoskeletal and Dermatological Science, School of Biological Sciences, Faculty of Biological Medicine and Health, University of Manchester, Manchester Academic Health Science Centre, Manchester, UK

**Keywords:** Prognostic markers, Depression

## Abstract

Vitamin D deficiency has been linked to various chronic pain conditions. However, randomized trials of vitamin D supplementation have had mixed results. In contrast, systematic reviews of randomized trials indicate a protective effect of vitamin D supplementation on depression. We undertake a Mendelian randomization investigation in UK Biobank, a study of UK residents aged 40-65 at recruitment. We perform linear and non-linear Mendelian randomization analyses for four outcomes: fibromyalgia, clinical fatigue, chronic widespread pain, and probable lifetime major depression. We use genetic variants from four gene regions with known links to vitamin D biology as instruments. In linear analyses, genetically-predicted levels of 25-hydroxyvitamin D [25(OH)D], a clinical marker of vitamin D status, were not associated with fibromyalgia (odds ratio [OR] per 10 nmol/L higher 25(OH)D 1.02, 95% confidence interval [CI] 0.93, 1.12), clinical fatigue (OR 0.99, 95% CI 0.94, 1.05), chronic widespread pain (OR 0.95, 95% CI 0.89, 1.02), or probable lifetime major depression (OR 0.97, 95% CI 0.93, 1.01). In non-linear analyses, an association was observed between genetically-predicted 25(OH)D levels and depression in the quintile of the population with the lowest 25(OH)D levels (OR 0.75, 95% CI 0.59, 0.94); associations were null in other strata. Our findings suggest that population-wide vitamin D supplementation will not substantially reduce pain or depression; however, targeted supplementation of deficient individuals may reduce risk of depression.

## Introduction

Vitamin D deficiency, defined as low levels of circulating 25-hydroxyvitamin D (25(OH)D) [1], has been linked to recurrent and chronic pain conditions [2]. Profound vitamin D deficiency is strongly linked to pain from osteomalacia, a bone weakening disorder [3]. Low vitamin D levels have also been suggested to play a role in the aetiology of chronic pain states through their influence on bone biology, autoimmune diseases, inflammation, or neuromuscular and other immunological influences on pain manifestation [4, 5]. Empirical evidence from observational studies indicates hypovitaminosis D to be highly prevalent in patients with chronic pain [6], fibromyalgia [7], and depression [8].

However, evidence for a direct causal link between low 25(OH)D levels and chronic pain conditions is incomplete. Randomized controlled trials of vitamin D as a treatment for chronic pain conditions, such as fibromyalgia, have generally shown no evidence of benefit, but most were limited in size and quality [9, 10]. In contrast, systematic reviews of randomized trials for depression [11], depressive symptoms [12, 13], and negative emotions [14] have indicated beneficial effects of vitamin D supplementation, although no evidence for an effect was observed in the large Vitamin D and Omega-3 Trial (VITAL) [15]. A potential explanation for this discrepancy is that vitamin D levels have a threshold effect on depression, which was not detected in the VITAL trial as many participants in the control arm had adequate vitamin D levels. Inverse associations have also been observed in observational epidemiological studies [16], although such investigations are vulnerable to bias from confounding and reverse causality: individuals with depression could have low vitamin D levels due to spending less time outdoors or changes in appetite and diet.

An alternative approach to randomized controlled trials that addresses the issues of confounding and reverse causality is Mendelian randomization. Genetic variants having a specific association with circulating 25(OH)D levels can be used as proxies for changes in 25(OH)D levels to assess the likelihood of a causal link between vitamin D and chronic pain conditions. As genetic variants are randomly distributed at conception, they are unlikely to be related to possible confounders [17]. In addition, chronic pain conditions cannot affect the genotype and so reverse causality is avoided [18]. As a result, Mendelian randomization analyses can provide more reliable insights into any potential causal relationship between vitamin D and chronic pain conditions compared with conventional observational studies. As the shape of the relationship between 25(OH)D serum levels and chronic pain conditions is unknown, and many observational vitamin D studies have shown a threshold effect [6]—where the disease risks are only related to the people with vitamin D deficiency – the standard Mendelian randomization approach which assumes linearity may overlook non-linear effects of 25(OH)D.

Given the uncertainty in the empirical literature as to the nature of the relationship between vitamin D serum levels and chronic pain conditions, we investigated evidence for a causal effect of 25(OH)D concentrations on chronic pain outcomes. We used individual-level data from the UK Biobank study to perform both linear and non-linear Mendelian randomization analyses. Linear analyses investigate the evidence for a causal effect in the population as a whole, and non-linear analyses investigate the evidence for a causal effect in strata of the population. We also considered depression as an additional outcome to validate trial findings and to assess the shape of any potential effect.

## Subjects and methods

### Study population

The UK Biobank cohort comprises around 500,000 participants aged 40–69 years at baseline, recruited between 2006–2010 at 22 assessment centres throughout the UK, and followed up for a variety of health conditions from their recruitment date until July 2019 or their date of death. Informed consent was obtained from all participants. The dataset includes genome-wide genotyping of baseline samples from all participants, results of clinical examinations, assays of biological samples, detailed information on self-reported health behaviour, and is supplemented by linkage with electronic health records such as hospital inpatient data, mortality data, and cancer registries [19].

We restricted our analysis to 333,025 unrelated participants of European ancestry that passed various quality control tests and had a valid 25(OH)D measurement. European ancestry was defined using self-reported ethnicity and genomic principal components as described previously [20]. We removed related individuals so that only one person in each family (defined as third-degree relatives or closer) was included in the analysis.

### Vitamin D measurement and classification

Concentrations of 25(OH)D in blood were measured using the DiaSorin Liaison immunoassay analyser. Measurements were mean-shifted to correspond to a measurement taken in October, in order to account for seasonal variability in 25(OH)D concentrations.

### Outcomes

We considered four outcomes: fibromyalgia, clinical fatigue, chronic widespread pain, and probable lifetime major depression.

Fibromyalgia was defined as ICD-9 code 729.1, ICD-10 code M79.7, or self-reported code (field 20002) 1542. Clinical fatigue was defined as ICD-9 code 780.7, ICD-10 code R53, or self-reported code 1482. Chronic widespread pain was defined as answering yes to the four questions “Have you had pain for more than 3 months?” relating to the neck or shoulder (field 3404), back (field 3571), hip (field 3414), and knees (field 3773).

Probable lifetime major depression was defined as the presence of four criteria [21]: 1) answering yes to the question “Looking back over your life, have you ever had a time when you were feeling depressed or down for at least a whole week?” or “Have you ever had a time when you were uninterested in things or unable to enjoy the things you used to for at least a whole week?”, 2) answering 2 weeks or more to the question “How many weeks was the longest period when you were feeling depressed or down?”, 3) answering 2 or more to the question “How many periods have you had when you were feeling depressed or down for at least a whole week?”, 4) answering yes to the question “Have you ever seen a general practitioner for nerves, anxiety, tension or depression?” or “Have you ever seen a psychiatrist for nerves, anxiety, tension or depression?”.

### Genetic variants

To minimize potential bias due to pleiotropy, we took genetic variants from four gene regions previously shown to be strongly associated with 25(OH)D levels, and implicated in the transport, metabolism, and synthesis of vitamin D [22]—*GC*, *DHCR7*, *CYP2R1*, and *CYP24A1*. Pleiotropy (also called “horizontal pleiotropy”) means that the genetic variants affect multiple traits on distinct causal pathways, which could lead to an association between the variants and the outcome that does not arise due to a causal effect of the exposure on the outcome. The *GC* gene encodes vitamin D binding protein. The product of the *DHCR7* gene converts 7-dehydrocholesterol to cholesterol, reducing 7-dehydrocholesterol available for conversion to previtamin D_3_ by solar radiation. The *CYP2R1* gene encodes vitamin D 25-hydroxylase, a regulator of 25(OH)D synthesis through 25-hydroxylation of vitamin D in the liver. The product of the *CYP24A1* gene inactivates the active form of vitamin D (1α25(OH)_2_D).

To maximize the variance explained by the genetic instrument, we selected variants for each gene region (coding region +/− 500 kilobases) associated with 25(OH)D concentrations in a multivariable regression model using a stepwise selection method [23]. This procedure allows variants to be moderately correlated, so long as they predict independent variation in the exposure. In total, 21 variants were included in the analysis (Supplementary Table 1). We constructed an allelic score (“focused score”) weighting variants by their conditional associations with 25(OH)D concentration. We also considered a score based on 71 genetic variants from across the genome (“genome-wide score”) previously demonstrated to be associated with 25(OH)D concentrations at a genome-wide level of statistical significance [24].

### Statistical analyses

#### Linear analyses

Linear Mendelian randomization estimates were calculated using the ratio method by dividing the genetic association with the outcome by the genetic association with 25(OH)D concentration and scaling the estimate to a 10 nmol/L difference in genetically-predicted 25(OH)D concentration. Genetic associations were estimated using logistic regression for disease outcomes and using linear regression for 25(OH)D concentrations. All regression models were adjusted for age at baseline, sex, centre, and ten genetic principal components of ancestry. Non-linear models were also adjusted for age-squared, age-by-sex, and age-squared-by-sex. Power calculations for the linear Mendelian randomization analyses were conducted using an online power calculator available at https://sb452.shinyapps.io/power/ [25].

#### Non-linear analyses

Non-linear Mendelian randomization estimates were performed by first dividing the population into five equal-sized strata using the doubly-ranked method [26]. The doubly-ranked method is implemented by first ordering individuals based on their level of the genetic score and dividing into pre-strata containing 50 individuals each, and then ordering individuals within each pre-stratum based on their level of the exposure. The lowest stratum consists of the ten individuals in each pre-stratum with the lowest levels of the exposure. The second lowest stratum consists of the 10 individuals in each pre-stratum with the second lowest levels of the exposure, and so on. We note this differs slightly from the original implementation of the method, which constructed pre-strata having the same size as the number of strata. By including more individuals in each pre-stratum, we more reliably select individuals with low levels of the exposure into the lowest strata, and hence differentiation between strata will be greater.

Estimates were obtained in each stratum as per the linear analyses. To stabilize estimates, we performed a bootstrap averaging approach: we removed a small number of participants (12 individuals) from the analysis at random and performed the doubly-ranked method using this dataset. We repeated this procedure 100 times, and then combined estimates using Rubin’s rules. Secondary analyses were performed by dividing the population into equal-sized strata using the residual method for untransformed and log-transformed values of 25(OH)D concentrations [27]. The doubly-ranked method has been recommended for use with 25(OH)D concentrations, as in this case the genetic effect on the exposure is not constant in the population; this assumption is made by the residual stratification method [28]. Additionally, we repeated all analyses dividing the population into three equal-sized strata rather than five strata. Further, to assess potential concerns with instrument validity in the non-linear analysis, we also considered age and sex as negative control outcomes [29] (analyses for age and sex were adjusted for centre and principal components only).

All statistical analyses were performed in R version 4.0.5.

### Ethics

UK Biobank ethical approval is provided by the UK Biobank research ethics committee and Human Tissue Authority research tissue bank. An independent Ethics and Governance Council oversees adherence to the Ethics and Governance Framework and provides advice on the interests of research participants and the general public in relation to UK Biobank. Informed consent was obtained from all subjects. The current study was approved by the UK Biobank (ref no. 98032). All methods were performed in accordance with the Declaration of Helsinki on ethical principles for medical research.

## Results

### Study population

**Fig. 1:**
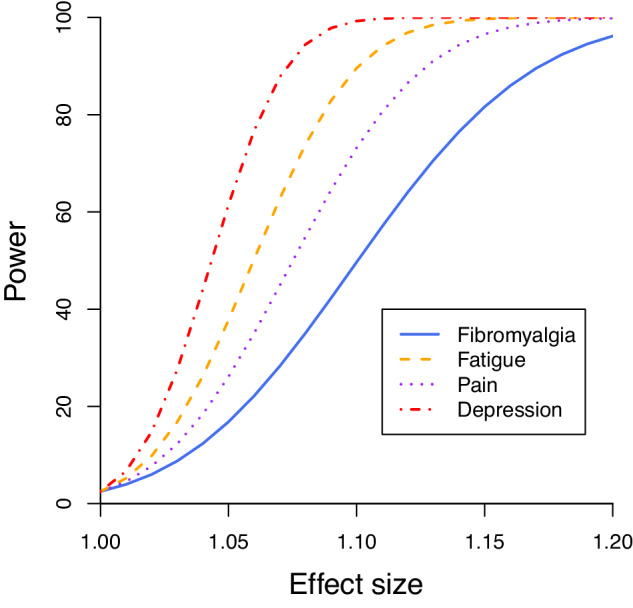
Power calculations for linear Mendelian randomization. Power to detect a causal effect of given effect size, representing an odds ratio per 10 nmol/L increase in genetically-predicted levels of 25(OH)D.

A summary of participant characteristics is presented in Table 1. The mean age of participants was 57.1 years, and 53.4% of participants were female. Mean 25(OH)D concentration (mean-shifted to correspond to an October measurement) was 55.4 nmol/L. 2424 participants (0.7%) had fibromyalgia, 6764 (2.0%) had clinical fatigue, 4279 (1.3%) had chronic widespread pain, and 13,125 (3.9%) had probable lifetime major depression. The genetic instrument explained 4.7% of the variance in 25(OH)D levels, corresponding to an F statistic of 745. Power calculations based on these parameters are presented in Fig. 1.

**Table 1:** Participant characteristics for the analytic sample from UK Biobank.

	UK Biobank
Participants	333 025
Age at baseline	57.1 ± 8.1
Sex	
Female	177 745 (53.4)
Male	155 280 (46.6)
25(OH)D concentration (nmol/L)	55.4 ± 19.3
Fibromyalgia cases	2424 (0.7)
Clinical fatigue cases	6764 (2.0)
Chronic widespread pain cases	4279 (1.3)
Probable lifetime major depression cases	13 125 (3.9)
BMI (kg/m^2^)	27·3 ± 4·8
SBP (mmHg)	137·5 ± 18·6
Smoking	
Current	34 085 (10·2)
Other	298 940 (89·8)
Diabetes	
Known history	15 822 (4·8)
No known history	317 203 (95·2)

Values represent mean ± standard deviation for continuous traits or N (%) for categorical traits. 25(OH)D concentrations are season-shifted to correspond to a measurement taken in autumn.

### Linear analyses

Linear Mendelian randomization estimates using the focused score, representing the odds ratio for the average association with the outcome for 10 nmol/L higher genetically-predicted 25(OH)D in the whole population, are provided in Table 2. Estimates were 1.02 (95% confidence interval [CI]: 0.93, 1.12; p = 0.65) for fibromyalgia, 0.99 (95% CI: 0.94, 1.05; p = 0.86) for clinical fatigue, 0.95 (95% CI: 0.89, 1.02; p = 0.18) for chronic widespread pain, and 0.97 (95% CI: 0.93, 1.01; p = 0.18) for probable lifetime major depression.

**Table 2:** Linear Mendelian randomization estimates in the overall population.

Outcome	Estimate	95% confidence interval	p-value
Fibromyalgia	1.02	0.93, 1.12	0.65
Clinical fatigue	0.99	0.94, 1.05	0.86
Chronic widespread pain	0.95	0.89, 1.02	0.18
Probable lifetime major depression	0.97	0.93, 1.01	0.18

Estimates represent odds ratios per 10 nmol/L higher genetically-predicted concentration of 25(OH)D in the overall population.

### Non-linear analyses

**Fig. 2:**
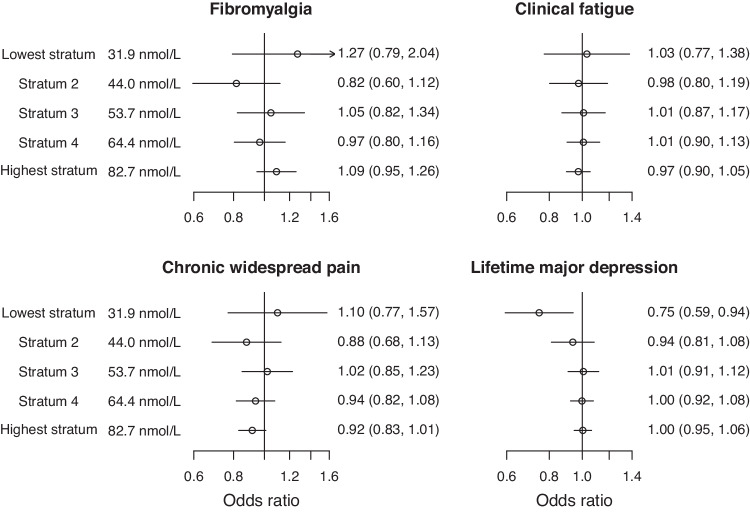
Non-linear Mendelian randomization estimates in five strata of the population. Concentrations are stratum-specific mean levels of 25(OH)D. Estimates (95% confidence intervals) represent odds ratios per 10 nmol/L higher genetically-predicted concentration of 25(OH)D in strata of the population defined using the doubly-ranked method.

Non-linear Mendelian randomization estimates using the focused score, representing the odds ratio for the average association with the outcome for 10 nmol/L higher genetically-predicted 25(OH)D in each stratum of the population, are provided in Fig. 2. As stratification is based on the exposure, the participants in each of the five strata (and hence the stratum-specific mean levels of 25(OH)D) are the same for each outcome. Associations were null in each stratum with the exception of probable lifetime major depression in the stratum with the lowest mean 25(OH)D levels (0.75, 95% CI: 0.59, 0.94; p = 0.013). A similar inverse association for probable lifetime major depression in this stratum was detected using the residual method for both untransformed and log-transformed 25(OH)D levels (Supplementary Table 2). We note that this finding would not be considered significant at a conventional multiply-corrected threshold accounting for the number of outcomes and strata (0.05/4/5 = 0.0025).

**Fig. 3:**
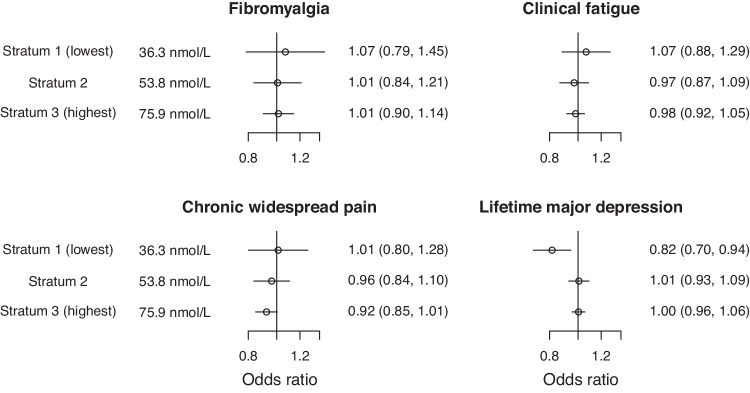
Non-linear Mendelian randomization estimates in three strata of the population. Concentrations are stratum-specific mean levels of 25(OH)D. Estimates (95% confidence intervals) represent odds ratios per 10 nmol/L higher genetically-predicted concentration of 25(OH)D in strata of the population defined using the doubly-ranked method.

Results were similar when dividing into three strata (Fig. 3, Supplementary Table 3). The association with probable lifetime major depression in the stratum with the lowest mean 25(OH)D levels was slightly attenuated but achieved a lower p-value due to the greater sample size (0.82, 95% CI: 0.70, 0.94; p = 0.006). Again, this finding would not be considered significant at a conventional multiply-corrected threshold accounting for the number of outcomes and strata (0.05/4/3 = 0.004).

**Fig. 4:**
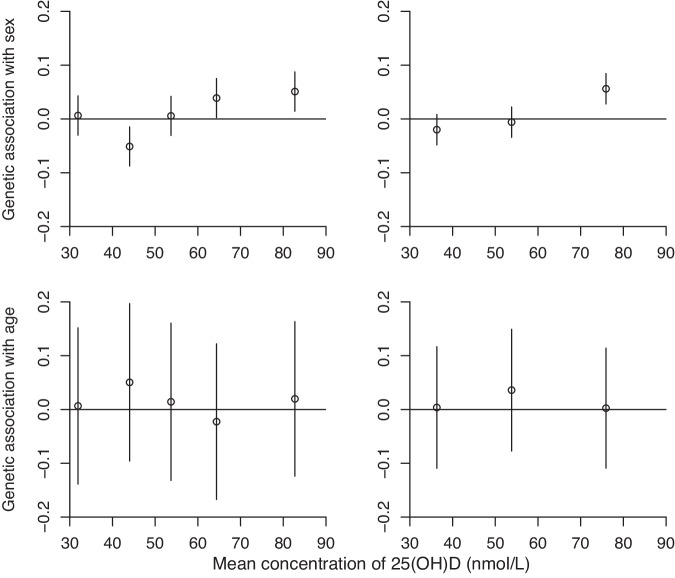
Stratum-specific associations with age and sex as negative controls. Concentrations are stratum-specific mean levels of 25(OH)D. Estimates (95% confidence intervals) represent association estimates with age (in years) and sex (log odds ratios) scaled to 10 nmol/L higher genetically-predicted concentration of 25(OH)D in five and three strata of the population defined using the doubly-ranked method.

Genetic associations with the negative controls age and sex are shown in Fig. 4. There are no clear associations with age, although there are slight associations with sex in some strata. This could be a symptom of differential selection into the sample, and indicates potential invalidity in the non-linear analyses, although adjustment for sex may mitigate any potential bias.

### Genome-wide score

We note that the genome-wide score is pleiotropic, and hence results using the genome-wide score should be regarded with caution. In particular, the score is associated with cholesterol levels; although the extent to which this may bias estimates is unclear. Linear Mendelian randomization estimates using the genome-wide score were 1.02 (95% CI: 0.92, 1.12; p = 0.73) for fibromyalgia, 1.02 (95% CI: 0.96, 1.08; p = 0.52) for clinical fatigue, 0.91 (95% CI: 0.85, 0.98; p = 0.011) for chronic widespread pain, and 0.96 (95% CI: 0.92, 1.00; p = 0.063) for probable lifetime major depression.

## Discussion

We conducted one-sample Mendelian randomization analyses in UK Biobank using both linear and non-linear methods. In analyses using the focused genetic score, we did not observe associations between genetically-predicted 25(OH)D and various outcomes in the population as a whole, suggesting that population shifts in vitamin D levels are unlikely to substantially decrease risk of fibromyalgia, clinical fatigue, chronic widespread pain, or probable lifetime major depression. However, we did observe a suggestive association with probable lifetime major depression in the stratum of the population with the lowest levels of 25(OH)D. This suggests that vitamin D supplementation may reduce risk of depression for those with low vitamin D levels.

Our findings may clarify evidence found in clinical trials for the effect of vitamin D supplementation on depression. While recent meta-analyses of randomized controlled trials of vitamin D supplementation have found evidence for reductions in depression and depressive symptoms [12–14], the VITAL trial did not observe any reduction in risk [15]. However, few individuals in the VITAL trial had deficient levels of vitamin D. Additionally, VITAL trial participants were allowed to continue taking low dose vitamin D supplements, even if they were assigned to the placebo arm of the trial. Our findings suggest that any benefit of vitamin D supplementation may be limited to those with low vitamin D levels and that supplementation beyond a threshold level may provide limited additional benefit. This corresponds to what has been seen in supplementation trials for acute respiratory tract infections, which have reported stronger evidence of risk reduction in those with 25(OH)D concentrations below 25 nmol/L [30]. Similarly, a previous systematic review of trials found that evidence of an inverse effect of vitamin D supplementation was limited to studies in those with deficient vitamin D levels at baseline [31]. This is also supported by research suggesting that vitamin D deficiency precedes the development of depression rather than being associated with its effects [32].

We did not observe evidence suggesting any effect of vitamin D supplementation on fibromyalgia, clinical fatigue, or chronic widespread pain, beyond a slight protective association in one stratum that may represent a chance finding. This may be due to lack of power, as the number of cases for these outcomes was less than that for depression. Another challenge is defining these outcomes, as these conditions are not always accurately captured in routinely-collected healthcare records. Our results concur with results from two clinical trials demonstrating no convincing benefit of vitamin D supplementation on pain [33, 34].

While our findings suggest that population-wide vitamin D supplementation will not substantially reduce risk of depression, they suggest vitamin D supplementation may reduce risk of depression for those with insufficient vitamin D levels. For any future clinical trials of vitamin D supplementation, this reinforces the importance of recruiting enough patients with vitamin D deficiency at baseline, so the trial has enough statistical power to detect an effect in deficient individuals. However, recruiting enough vitamin D deficient individuals into such a trial to attain adequate power, and ensuring that those assigned to the placebo group do not self-medicate, may prove challenging in practice.

A number of different biological mechanisms might explain the direct relationship observed between low levels of circulating 25(OH)D and probable lifetime major depression. Inflammation is proposed to play a causal role in the development of depression [35, 36], and vitamin D has been observed to play an anti-inflammatory role in neurological functioning by preventing oxidative damage to nervous tissue [37]. It has also been suggested in several randomized controlled trials that higher vitamin D levels result in lower levels of systemic inflammatory cytokines [38]. Mechanisms underpinning the inflammatory hypothesis for depression are an area of ongoing research [39]. Recent studies in mice also indicate that a lack of vitamin D might influence microbiome diversity related to mood disorders [40], in line with recent research into the role of gut microbiome in depression [41]. Finally, in vitro, work has also suggested a connection between vitamin D and the hypothalamic–pituitary–adrenal (HPA) axis via influence on the glucocorticoid receptors [42], which has a known role in the development of depression [43]. Given the number of biological pathways through which vitamin D levels might play a role in risk of depression, this may explain the associations we found between lower genetically-predicted 25(OH)D levels and probable lifetime major depression but not chronic pain conditions.

Our investigation has strengths in the Mendelian randomization design, large sample size, and biologically-driven choice of genetic variants. However, there are also potential limitations. Our analysis was restricted to individuals with European ancestry. This limits the validity and applicability of the findings, particularly as individuals with darker skin have lower 25(OH)D levels. Mendelian randomization analyses make the assumption that the only causal pathway from the genetic variants to the outcome is via the exposure. While the four gene regions chosen for this study have strong biological links to vitamin D, this assumption cannot be tested empirically. Further, even if the Mendelian randomization assumptions are satisfied, genetic variants may influence 25(OH)D concentrations in a different way to dietary supplementation or other clinical interventions. We used the doubly-ranked method for non-linear Mendelian randomization, which is more robust than the residual method, but some residual bias owing to variability in the genetic effects on the exposure may remain [28]. Our outcome definitions are likely to have missed some cases; we only identified around 4% of UK Biobank participants as having depression, whereas lifetime prevalence of major depression is around 10-20% according to general population studies [44]. Misclassification of cases as controls generally introduces a bias towards the null, so the genetic associations observed in our analysis are likely to be conservative and hence may underestimate the true impact on disease risk.

Concerns have been raised about the potential for bias in non-linear Mendelian randomization due to associations with age and sex in strata [29]. Such associations are a sign of instrument invalidity, as it is impossible for vitamin D to affect age or sex. In our example, we saw slight associations within some strata for sex, but not age. These associations could arise due to selection bias in the UK Biobank dataset, or violation of the rank-preserving assumption made by the doubly-ranked method [45]. Adjustment for age and sex could potentially mitigate this bias. It is notable that most non-linear Mendelian randomization analyses using the doubly-ranked method for vitamin D (both those in this manuscript, and those published previously) have given null results [23]. If stratification led to pervasive bias, then we would expect non-null estimates for all outcomes. Additionally, the non-null association in low vitamin D individuals is for the outcome with the strongest evidence of an effect of vitamin D supplementation from randomized trials. Hence, the specificity of our finding for depression gives us confidence that this result is not driven by a bias mechanism that would be expected to affect all analyses.

In conclusion, we found evidence for an effect of vitamin D on depression in individuals with low vitamin D status, but no evidence of an effect at any level of 25(OH)D for other pain outcomes.

### Supplementary information

Supplementary Tables

## Data Availability

Software code for performing the analyses in the paper is available at https://github.com/amymariemason/SUMnlmr.

## Data availability

UK Biobank data is available on reasonable request to any bona fide researcher for health-related research that is in the public interest. See https://www.ukbiobank.ac.uk/enable-your-research for more details.

## References

[1] Holick MF (2009). Vitamin D status: measurement, interpretation, and clinical application. Ann Epidemiol.

[2] Wu Z, Malihi Z, Stewart AW, Lawes CMM, Scragg R (2018). The association between vitamin D concentration and pain: a systematic review and meta-analysis. Public Health Nutr.

[3] Christodoulou S, Goula T, Ververidis A, Drosos G (2013). Vitamin D and bone disease. Biomed Res Int.

[4] Wintermeyer E, Ihle C, Ehnert S, Stöckle U, Ochs G, de Zwart P (2016). Crucial role of vitamin D in the musculoskeletal system. Nutrients.

[5] Eyles DW, Burne TH, McGrath JJ (2013). Vitamin D, effects on brain development, adult brain function and the links between low levels of vitamin D and neuropsychiatric disease. Front Neuroendocrinol.

[6] Helde-Frankling M, Björkhem-Bergman L (2017). Vitamin D in pain management. Int J Mol Sci.

[7] Karras S, Rapti E, Matsoukas S, Kotsa K (2016). Vitamin D in fibromyalgia: a causative or confounding biological interplay?. Nutrients.

[8] Milaneschi Y, Hoogendijk W, Lips P, Heijboer AC, Schoevers R, van Hemert AM (2014). The association between low vitamin D and depressive disorders. Mol Psychiatry.

[9] Shipton EE, Shipton EA (2015). Vitamin D deficiency and pain: clinical evidence of low levels of vitamin D and supplementation in chronic pain states. Pain Ther.

[10] Straube S, Derry S, Moore RA, McQuay HJ. Vitamin D for the treatment of chronic painful conditions in adults. Cochrane Database Syst Rev. 2010:Cd007771. 10.1002/14651858.CD007771.pub2 PMC4170888 20091647

[11] Vellekkatt F, Menon V (2019). Efficacy of vitamin D supplementation in major depression: a meta-analysis of randomized controlled trials. J Postgrad Med.

[12] Musazadeh V, Keramati M, Ghalichi F, Kavyani Z, Ghoreishi Z, Alras KA (2023). Vitamin D protects against depression: Evidence from an umbrella meta-analysis on interventional and observational meta-analyses. Pharmacol Res.

[13] Mikola T, Marx W, Lane MM, Hockey M, Loughman A, Rajapolvi S (2023). The effect of vitamin D supplementation on depressive symptoms in adults: A systematic review and meta‐analysis of randomized controlled trials. Crit Rev Food Sci Nutr.

[14] Cheng Y-C, Huang Y-C, Huang W-L (2020). The effect of vitamin D supplement on negative emotions: a systematic review and meta-analysis. Depression Anxiety.

[15] Okereke OI, Reynolds CF, Mischoulon D, Chang G, Vyas CM, Cook NR (2020). Effect of long-term vitamin D3 supplementation vs placebo on risk of depression or clinically relevant depressive symptoms and on change in mood scores: a randomized clinical trial. JAMA.

[16] Anglin RE, Samaan Z, Walter SD, McDonald SD (2013). Vitamin D deficiency and depression in adults: systematic review and meta-analysis. Br J Psychiatry.

[17] Davey Smith G, Hemani G (2014). Mendelian randomization: genetic anchors for causal inference in epidemiological studies. Hum Mol Genet.

[18] Burgess S, Swanson SA, Labrecque JA (2021). Are Mendelian randomization investigations immune from bias due to reverse causation?. Eur J Epidemiol.

[19] Sudlow C, Gallacher J, Allen N, Beral V, Burton P, Danesh J (2015). UK Biobank: an open access resource for identifying the causes of a wide range of complex diseases of middle and old age. PLOS Med.

[20] Astle WJ, Elding H, Jiang T, Allen D, Ruklisa D, Mann AL (2016). The allelic landscape of human blood cell trait variation and links to common complex disease. Cell.

[21] Smith DJ, Nicholl BI, Cullen B, Martin D, Ul-Haq Z, Evans J (2013). Prevalence and characteristics of probable major depression and bipolar disorder within UK biobank: cross-sectional study of 172,751 participants. PLoS One.

[22] Mokry LE, Ross S, Ahmad OS, Forgetta V, Smith GD, Leong A (2015). Vitamin D and risk of multiple sclerosis: a Mendelian randomization study. PLOS Med.

[23] Sofianopoulou E, Kaptoge SK, Afzal S, Jiang T, Gill D, Gundersen TE (2023). Estimating dose-response relationships for vitamin D with coronary heart disease, stroke, and all-cause mortality: observational and Mendelian randomisation analyses. Lancet Diab Endocrinol.

[24] Manousaki D, Mitchell R, Dudding T, Haworth S, Harroud A, Forgetta V (2020). Genome-wide association study for vitamin D levels reveals 69 independent loci. Am J Hum Genet.

[25] Burgess S (2014). Sample size and power calculations in Mendelian randomization with a single instrumental variable and a binary outcome. Int J Epidemiol.

[26] Tian H, Mason AM, Liu C, Burgess S (2023). Relaxing parametric assumptions for non-linear Mendelian randomization using a doubly-ranked stratification method. PLOS Genet.

[27] Burgess S, Davies NM, Thompson SG (2014). Instrumental variable analysis with a nonlinear exposure-outcome relationship. Epidemiology.

[28] Burgess S (2023). Violation of the constant genetic effect assumption can result in biased estimates for non-linear Mendelian randomization. Hum Heredity.

[29] Hamilton FW, Hughes DA, Spiller W, Tilling K, Davey Smith G. Non-linear mendelian randomization: detection of biases using negative controls with a focus on BMI, Vitamin D and LDL cholesterol. Eur J Epidemiol. 2024. https://pubmed.ncbi.nlm.nih.gov/38789826/. 10.1007/s10654-024-01113-9 PMC11219394 38789826

[30] Martineau AR, Jolliffe DA, Hooper RL, Greenberg L, Aloia JF, Bergman P (2017). Vitamin D supplementation to prevent acute respiratory tract infections: systematic review and meta-analysis of individual participant data. Br Med J.

[31] Spedding S (2014). Vitamin D and depression: a systematic review and meta-analysis comparing studies with and without biological flaws. Nutrients.

[32] Ronaldson A, Arias de la Torre J, Gaughran F, Bakolis I, Hatch SL, Hotopf M (2022). Prospective associations between vitamin D and depression in middle-aged adults: findings from the UK Biobank cohort. Psychol Med.

[33] Soens MA, Sesso HD, Manson JE, Fields KG, Buring JE, Lee I-M (2024). The effect of vitamin D and omega-3 fatty acid supplementation on pain prevalence and severity in older adults: a large-scale ancillary study of the Vitamin D and OmegA-3 triaL (VITAL). Pain.

[34] Wu Z, Camargo CA, Malihi Z, Bartley J, Waayer D, Lawes CMM (2018). Monthly Vitamin D supplementation, pain, and pattern of analgesic prescription: secondary analysis from the randomized, double-blind, placebo-controlled Vitamin D Assessment study. Pain.

[35] Miller AH, Raison CL (2016). The role of inflammation in depression: from evolutionary imperative to modern treatment target. Nat Rev Immunol.

[36] Ye Z, Kappelmann N, Moser S, Davey Smith G, Burgess S, Jones PB (2021). Role of inflammation in depression and anxiety: Tests for disorder specificity, linearity and potential causality of association in the UK Biobank. eClinicalMedicine.

[37] Wrzosek M, Łukaszkiewicz J, Wrzosek M, Jakubczyk A, Matsumoto H, Piątkiewicz P (2013). Vitamin D and the central nervous system. Pharmacol Rep.

[38] Cannell JJ, Grant WB, Holick MF (2014). Vitamin D and inflammation. Dermatoendocrinology.

[39] Berk M, Williams LJ, Jacka FN, O’Neil A, Pasco JA, Moylan S (2013). So depression is an inflammatory disease, but where does the inflammation come from?. BMC Med.

[40] Jin D, Wu S, Zhang YG, Lu R, Xia Y, Dong H (2015). Lack of Vitamin D receptor causes dysbiosis and changes the functions of the murine intestinal microbiome. Clin Ther.

[41] Winter G, Hart RA, Charlesworth RPG, Sharpley CF (2018). Gut microbiome and depression: what we know and what we need to know. Rev Neurosci.

[42] Obradovic D, Gronemeyer H, Lutz B, Rein T (2006). Cross-talk of Vitamin D and glucocorticoids in hippocampal cells. J Neurochem.

[43] Pariante CM, Lightman SL (2008). The HPA axis in major depression: classical theories and new developments. Trends Neurosci.

[44] Lim GY, Tam WW, Lu Y, Ho CS, Zhang MW, Ho RC (2018). Prevalence of Depression in the Community from 30 Countries between 1994 and 2014. Sci Rep.

[45] Burgess S. Towards more reliable non-linear Mendelian randomization studies. Eur J Epidemiol. 2024. https://pubmed.ncbi.nlm.nih.gov/38789825/. 10.1007/s10654-024-01121-9 PMC7616246 38789825

