# Supplementary material for: Vitamin D, chronic pain, and depression: linear and non-linear Mendelian randomization analyses
Source: Transl Psychiatry. 2024 Jul 4;14:274. doi: 10.1038/s41398-024-02997-7 (PMC11224391; doi:10.1038/s41398-024-02997-7)
Supplement: Supplementary file 1 — Supplementary Tables [file 41398_2024_2997_MOESM1_ESM.pdf]

Supplementary Table 1: List of genetic variants for allelic score

Chromosome: Position (hg19)	rsID	Effect allele	Other allele	Conditional association with 25(OH)D (nmol/L)
4:72617775	rs1352846	G	A	0.172
4:72618334	rs7041	C	A	-0.045
4:72634343	rs4694431	T	C	-0.034
4:72770563	rs139148694	GTGCTTTTATCAA	G	0.028
11:14339328	rs16913816	A	G	-0.031
11:14900931	rs117913124	A	G	0.503
11:14912573	rs117576073	T	G	0.246
11:14913575	rs12794714	A	G	0.139
11:14913645	rs202122669	A	G	-0.615
11:14913900	rs187639972	C	G	-0.360
11:14941652	rs117115472	G	C	0.148
11:71157867	rs139168803	A	G	-0.188
11:71158672	rs12573951	G	A	-0.045
11:71161063	rs7928249	G	A	-0.131
11:71180762	rs549000212	A	C	-0.364
11:71290740	rs4081429	C	A	0.017
20:52714706	rs6123359	G	A	-0.026
20:52731402	rs6127099	T	A	0.013
20:52735238	rs35870583	GT	G	0.027
20:52737123	rs2585442	G	C	-0.025
20:52788925	rs2762942	A	G	-0.053

Supplementary Table 2: Non-linear Mendelian randomization estimates for probable lifetime major depression from different methods with five strata.

	Doubly-ranked method		Residual method (untransformed exposure)		Residual method (log-transformed exposure)	
Stratum	Estimate	95% CI	Estimate	95% CI	Estimate	95% CI
1 (lowest)	0.75	0.59, 0.94	0.86	0.75, 0.99	0.91	0.82, 1.01
2	0.94	0.81, 1.08	0.94	0.85, 1.03	0.94	0.85, 1.03
3	1.01	0.91, 1.12	0.97	0.88, 1.06	1.02	0.92, 1.12
4	1.00	0.92, 1.08	1.00	0.92, 1.10	0.97	0.88, 1.07
5 (highest)	1.00	0.95, 1.06	1.01	0.93, 1.09	1.02	0.92, 1.12

Estimates (95% confidence intervals) represent odds ratios per 10 nmol/L higher genetically-predicted concentration of 25(OH)D (for doubly-ranked method and residual method with untransformed exposure), or per 20% higher genetically-predicted concentration of 25(OH)D (for residual method with log-transformed exposure).

Supplementary Table 3: Non-linear Mendelian randomization estimates for probable lifetime major depression from different methods with three strata.

	Doubly-ranked method		Residual method (untransformed exposure)		Residual method (log-transformed exposure)	
Stratum	Estimate	95% CI	Estimate	95% CI	Estimate	95% CI
1 (lowest)	0.82	0.70, 0.94	0.85	0.76, 0.95	0.91	0.83, 0.99
2	1.01	0.93, 1.09	1.02	0.95, 1.09	1.00	0.93, 1.08
3 (highest)	1.00	0.96, 1.06	0.98	0.93, 1.04	0.99	0.92, 1.06

Estimates (95% confidence intervals) represent odds ratios per 10 nmol/L higher genetically-predicted concentration of 25(OH)D (for doubly-ranked method and residual method with untransformed exposure), or per 20% higher genetically-predicted concentration of 25(OH)D (for residual method with log-transformed exposure).
